# Prolonged storage reduces viability of *Peptacetobacter (Clostridium) hiranonis* and core intestinal bacteria in fecal microbiota transplantation preparations for dogs

**DOI:** 10.3389/fmicb.2024.1502452

**Published:** 2025-01-07

**Authors:** Bruna Correa Lopes, Jonathan Turck, M. Katherine Tolbert, Paula R. Giaretta, Jan S. Suchodolski, Rachel Pilla

**Affiliations:** ^1^Gastrointestinal Laboratory, Department of Small Animal Clinical Sciences, Texas A&M University, College Station, TX, United States; ^2^Department of Veterinary Pathology, Hygiene and Public Health, University of Milan, Milan, Italy

**Keywords:** *Clostridium hiranonis*, dysbiosis, canine, cryoprotectant, PMA, bacterial culture, lyophilization, bile acid metabolism

## Abstract

**Introduction:**

Fecal microbiota transplantation (FMT) has been described useful as an adjunct treatment for chronic enteropathy in dogs. Different protocols can be used to prepare and store FMT preparations, however, the effect of these methods on microbial viability is unknown. We aimed (1) to assess the viability of several core intestinal bacterial species by qPCR and (2) to assess *Peptacetobacter* (*Clostridium*) *hiranonis* viability through culture to further characterize bacterial viability in different protocols for FMT preparations.

**Methods:**

Bacterial abundances were assessed in feces from six healthy dogs by qPCR after propidium monoazide (PMA-qPCR) treatment for selective quantitation of viable bacteria. Conservation methods tested included lyophilization (stored at 4°C and at −20°C) and freezing with glycerol-saline solution (12.5%) and without any cryoprotectant (stored at −20°C). Additionally, the abundance of *P. hiranonis* was quantified using bacterial culture.

**Results:**

Using PMA-qPCR, the viability of *Faecalibacterium*, *Escherichia coli*, *Streptococcus*, *Blautia*, *Fusobacterium*, and *P. hiranonis* was reduced in lyophilized fecal samples kept at 4°C and −20°C up to 6 months (*p* < 0.05). In frozen feces without cryoprotectant, only *Streptococcus* and *E. coli* were not significantly reduced for up to 3 months (*p* > 0.05). Lastly, no differences were observed in the viability of those species in glycerol-preserved samples up to 6 months (p > 0.05). When using culture to evaluate the viability of *P. hiranonis*, we observed that *P. hiranonis* abundance was lower in lyophilized samples kept at 4°C than −20°C; and *P. hiranonis* abundance was higher in glycerol-preserved samples for up to 6 months than in samples preserved without glycerol for up to 3 months. Moreover, the highest abundance of *P. hiranonis* was observed in glycerol-preserved feces. After 3 months, *P. hiranonis* was undetectable by culture in 83% (5/6) of the frozen samples without glycerol.

**Discussion:**

While the lyophilization procedure initially reduced *P. hiranonis* abundance, *P. hiranonis* viability was stable thereafter for up to 6 months at −20°C. The higher bacterial viability detected in fecal samples preserved with glycerol confirms the use of this cryoprotectant as a reliable method to keep bacteria alive in the presence of fecal matrix for FMT purposes.

## 1 Introduction

Fecal microbiota transplantation (FMT) is defined as the transplantation of feces from a healthy donor to a recipient, aiming to modulate the recipient’s intestinal microbiota and potentially confer beneficial effects (Cammarota et al., 2017; Chaitman and Gaschen, 2021; Borody and Khoruts, 2011). In humans, FMT has been notably successful in treating *Clostridioides difficile* enteric infection and it has been pointed out as an efficient treatment for recurring *C. difficile* infections (Mullish et al., 2018; Costello et al., 2015; Monaghan et al., 2019; Bishop and Tiruvoipati, 2022). In veterinary medicine, FMT is an adjunct treatment for chronic and acute diarrhea, and it can be used in the recovery of companion animals with antibiotic-induced dysbiosis (Chaitman and Gaschen, 2021; Chaitman et al., 2020; Toresson et al., 2023; Collier et al., 2022). FMT has also been investigated as a tool to improve clinical signs in a myriad of diseases in humans and animal models, from obesity to cancer, with varying results (Bäckhed et al., 2004; Napolitano and Covasa, 2020; Ting et al., 2022).

The exact mechanism of how FMT works is currently unknown: live bacteria, bacterial products, metabolites, or a combination of those may be related to the success of FMT (Borody and Khoruts, 2011; Scaldaferri et al., 2016; Segal et al., 2020). Khoruts and Sadowsky postulated that the effectiveness of FMT in humans may be linked to or dependent on the ability of beneficial bacteria from the donor to establish themselves in the recipient’s intestine, a process known as colonization or engraftment (Khoruts and Sadowsky, 2016). For colonization to occur, the viability of those microorganisms linked to healthy status in donors must be conserved during the FMT procedure. In addition, fecal metabolites may impact the overall intestine health by signaling downstream pathways relevant to certain diseases’ pathogenesis (Suez and Elinav, 2017; Ott et al., 2017; Butzner et al., 1996). Modulation of certain metabolites present in the gut such as the provision of butyrate and modification of the intestinal bile acid pool, including high fecal concentration of secondary bile acid, have been correlated to gastrointestinal health improvement in animal models (Butzner et al., 1996; Buffie et al., 2015).

The quantitative evaluation of bacterial viability in FMT preparations can be assessed through classical bacteriology (culture) or molecular biology (qPCR and sequencing). When assessing viability through bacterial culture, identifying all different cultivable bacterial species within the fecal sample can be challenging (Oliver, 2005), especially for those species with unremarkable colony morphology; however, culture remains the gold standard for evaluating the viability of certain bacterial species (Costello et al., 2015). On the other hand, when assessing viability using molecular biology tools, important limitations arise in distinguishing between DNA from live bacteria, “free-floating” DNA, and DNA from viable but nonculturable bacteria within a sample. The use of propidium monoazide (PMA) has been described in human and veterinary literature as a potential methodology to assess bacterial viability (Papanicolas et al., 2019; Fouhy et al., 2015; Lee and Bae, 2018). Despite FMT being often used as a treatment in veterinary and human medicine, there is limited literature on the viability of bacteria in FMT preparations, regardless of the method used for bacterial viability assessment (Costello et al., 2015; Papanicolas et al., 2019; Fouhy et al., 2015; Costello et al., 2017).

Although many bacterial species are transplanted with FMT, only a few species have been consistently correlated with maintaining a healthy intestinal microbiome in dogs, such as *Faecalibacterium*, *Fusobacterium*, *Turicibacter*, *Blautia*—which are short-chain fatty acids (SCFA) producers (Vital et al., 2014; Liu et al., 2021; Lynch et al., 2023)—and *Peptacetobacter* (*Clostridium*) *hiranonis*, the main species responsible for the conversion of primary into secondary bile acid in dogs and cats through the 7α-dehydroxylation pathway (AlShawaqfeh et al., 2017; Hirano et al., 1981; Correa Lopes et al., 2024). The SCFAs, including acetate, propionate, and butyrate, play several roles in host metabolism, with butyrate being the primary energy source for colonocytes in the gut (Deleu et al., 2021). In addition, the metabolism of bile acids is an important pathway modulated by intestinal microbiota, regulating lipid and glucose metabolism, energy production, and inflammatory signaling (Chiang, 2013). Dysmetabolism of bile acids and reduction of *P. hiranonis* have been linked to severe dysbiosis (Sung et al., 2023; Pilla et al., 2020; Manchester et al., 2019) and intestinal colonization by pathogenic species such as *C. difficile* in dogs (Werner et al., 2023; Sulaiman et al., 2024).

In the context of bile acid metabolism, the gut microbiota plays a crucial role in two essential metabolic functions related to bile acids that escape enterohepatic reabsorption: deconjugation and conversion of primary to secondary fecal unconjugated bile acids (Hofmann, 2009; Vinithakumari et al., 2021). While many bacterial species deconjugate glycine- and taurine-conjugated bile acids through bile salt hydrolase (Song et al., 2019; Jones et al., 2008), only a few bacterial species have been identified as responsible for converting unconjugated primary bile acids—cholic acid and chenodeoxycholic acid—into unconjugated secondary bile acids—deoxycholic acid and lithocholic acid, respectively (Cai et al., 2022; Vital et al., 2019). Among these bacteria is *P. hiranonis*, which possesses the bile acid-inducible operon encoding enzymes responsible for converting bile acids through the 7α-dehydroxylation pathway (Hirano et al., 1981; Hirano and Masuda, 1982; Doerner et al., 1997; Wise and Cummings, 2022; Ridlon et al., 2020). *C. hiranonis*, recently renamed *P. hiranonis* (Chen et al., 2020), is an anaerobic, spore-forming, and gram-positive bacteria (Chen et al., 2020; Kitahara et al., 2001). In dogs, *P. hiranonis* is described as a biomarker for gastrointestinal functionality and is closely linked to maintaining a balanced gastrointestinal health (Félix et al., 2022; Suchodolski, 2022). *P. hiranonis* plays a pivotal role in the conversion of primary to secondary fecal unconjugated bile acids and is the main species with this ability within canine and feline gastrointestinal microbiomes (Correa Lopes et al., 2024; Pilla et al., 2020; Vinithakumari et al., 2021; Giaretta et al., 2018).

Changes in gastrointestinal microbiota composition and abundance of *P. hiranonis* induced by antibiotic use can disrupt bile acid metabolism (Chaitman et al., 2020; Manchester et al., 2019; Blake et al., 2019). Dysbiosis and dysmetabolism of bile acids have been linked to chronic enteropathy in dogs (Guard et al., 2019; Vecchiato et al., 2023) and other chronic inflammatory diseases in humans (Prawitt et al., 2011; Wang et al., 2017). While the link between intestinal healthy microbiota and *P. hiranonis*, *Faecalibacterium*, *Fusobacterium*, *Turicibacter*, and *Blautia* in dogs has been outlined (Pilla et al., 2020; Suchodolski, 2022; Guard et al., 2019), our understanding of how their viability is affected by the FMT manufacturing process remains not completely elucidated. This study aims: (1) to evaluate the effect of lyophilization, freezing, and storage in different temperatures on bacterial survival by assessing the viability of core bacteria *Faecalibacterium*, *Fusobacterium*, *Turicibacter*, *Blautia*, *P. hiranonis*, *Streptococcus*, and *Escherichia coli* as well as total bacteria in fecal samples of potential FMT donors using PMA-qPCR; and (2) to evaluate the effect of the previously mentioned methods of FMT conservation on the viability of *P. hiranonis* using bacterial culture.

## 2 Materials and methods

### 2.1 Sampling, conservation methods, and time points

Fecal samples were collected from six clinically healthy dogs undergoing screening as potential FMT donors (Supplementary Table S1). Fecal samples were collected immediately post-defecation and transported to the laboratory in an insulated cooler using ice packs. All samples were processed within 24 h of defecation. Samples were divided into multiple vials for lyophilization, freezing with glycerol, and freezing without any cryoprotectants. The lyophilization process began by freezing the samples for 1 h at −80°C, followed by overnight drying. After lyophilization, the aliquoted samples were stored at 4°C and − 20°C for further analysis. For glycerol-preserved samples, we followed the protocol described by Chen et al. (2021), which involved adding 10 g of saline with glycerol (final concentration of glycerol: 12.5%) to 1 g of stool. These samples were kept frozen at −20°C until further analysis. Finally, aliquots without cryoprotectants were stored at −20°C. Throughout the study, samples preserved at both 4°C and − 20°C were evaluated by culture and PMA-qPCR at specific time points: fresh feces, and after 1 week, 1, 3, and 6 months (Table 1). Unfortunately, there were not enough feces for the evaluation of the last time point for fecal samples conserved without cryoprotectants.

**Table 1:** Methods of conservation and temperature of storage to preserve bacterial viability in fecal samples from healthy dogs evaluated at different time points by culture and PMA-qPCR.

Methods of FMT preparation	Temperature of storage	Time points
Lyophilization	4°C and −20°C	F, 1W, 1M, 3M, and 6M
Freezing with glycerol	–20°C	F, 1W, 1M, 3M, and 6M
Freezing without cryoprotectant	–20°C	F, 1M, and 3M

F, fresh fecal sample; 1W, 1 week; 1M, 1 month; 3M, 3 months; 6M, 6 months.

### 2.2 Propidium monoazide treatment of fecal samples for viability qPCR

Fecal samples were 10-fold diluted in phosphate-buffered saline (0.008 M sodium phosphate, 0.002 M potassium phosphate, 0.14 M sodium chloride, 0.01 M potassium chloride, pH 7.4, 500 mL; Pierce™, IL), vortexed for 5 min, and incubated on an ice rack for 15 min. After incubation, 500 μL from the supernatant was added to a new tube and 5 μL of propidium monoazide (2.5 mM) was added. Samples were mixed for 10 min (60 rpm) and exposed to light to cross-link PMAxx to the DNA for 15 min. After centrifugation at 5000 × g for 10 min, pelleted cells were re-suspended and used for DNA extraction (Nye et al., 2023).

### 2.3 DNA extraction and qPCR assays

The DNA from fecal samples were isolated using MoBio Power Soil Kit (MoBio Laboratories), following the manufacturer’s instructions. The qPCR assay was performed using SsoFast EvaGreen® Supermix (Bio-Rad Laboratories) as described by AlShawaqfeh et al. (2017) and Sung et al. (2022). Briefly, the concentration of the extracted DNA was assessed using a spectrophotometer (NanoDrop 1,000; Thermo Scientific) and normalized to 5 ng/μl. For the PCR reaction, 2 μL normalized DNA (5 ng/μl) was added to 5 μL SsoFast EvaGreen Supermix (Bio-Rad Laboratories), along with 0.4 μL each of forward and reverse primers (400 nM) (Supplementary Table S2), and 2.2 μL DNA-free water. The Bio-Rad CFX384 Touch Thermal Cycler (Bio-Rad Laboratories) was used in this study. The cycling protocol is described as follows: initial denaturation at 98°C for 2 min; 35 cycles with denaturation at 98°C for 3 s; and annealing for 3 s. All samples were analyzed in duplicate (CFX MaestroTM 1.1 Software) and the average of the two results was used for the analysis. The quantification of the log amount of DNA (number of copies) per 10 ng of total isolated DNA was based on a standard curve. In summary, a 10-fold dilution series of purified plasmids containing the sequences for each bacterial target was used to construct the standard curve for DNA quantification. The amplicon length, melting peak temperature, efficiency of the qPCR assay, and the coefficient of determination (R2) of the calibration curve were previously described (AlShawaqfeh et al., 2017; Sung et al., 2022).

### 2.4 *Peptacetobacter hiranonis* culture, identification and quantification

The viability of *P. hiranonis* was assessed by bacterial culture. Fecal samples were serially 10-fold diluted in phosphate-buffered saline (0.008 M sodium phosphate, 0.002 M potassium phosphate, 0.14 M sodium chloride, 0.01 M potassium chloride, pH 7.4, 500 mL; Pierce™, IL), and plated onto Brucella blood agar plates (Anaerobic Systems, CA). For the quantification of *P. hiranonis,* all colonies morphologically compatible with *P. hiranonis* (Supplementary Figure S1) were collected for further identification using molecular methods. DNA extraction from each colony was performed as described by Dashti et al. (2009), and confirmation of *P. hiranonis* by qPCR was conducted following the protocol described by AlShawaqfeh et al. (2017).

### 2.5 Statistical analysis

Shapiro–Wilk’s test was employed for the normality assessment of the data. Comparison between time points (fresh fecal sample, 1 week, 1, 3, and 6 months) and different FMT preparation methods (lyophilization stored at 4°C and −20°C, feces preserved with and without glycerol stored at −20°C) were evaluated using Repeated Measures One-way ANOVA and Dunnett’s multiple comparison test. Spearman’s test was employed to assess the correlation between the abundance of *P. hiranonis* assessed by PMA-qPCR, expressed in log DNA, and viability of *P. hiranonis* assessed by bacterial culture, expressed in log CFU/g of feces, through the time points and different methods of FMT preparations. The statistical analysis was performed in GraphPad Prism (Version 9.4.1). A *p*-value of less than 0.05 was considered statistically significant.

## 3 Results

### 3.1 Bacterial viability assessed by PMA-qPCR

**Figure 1:**
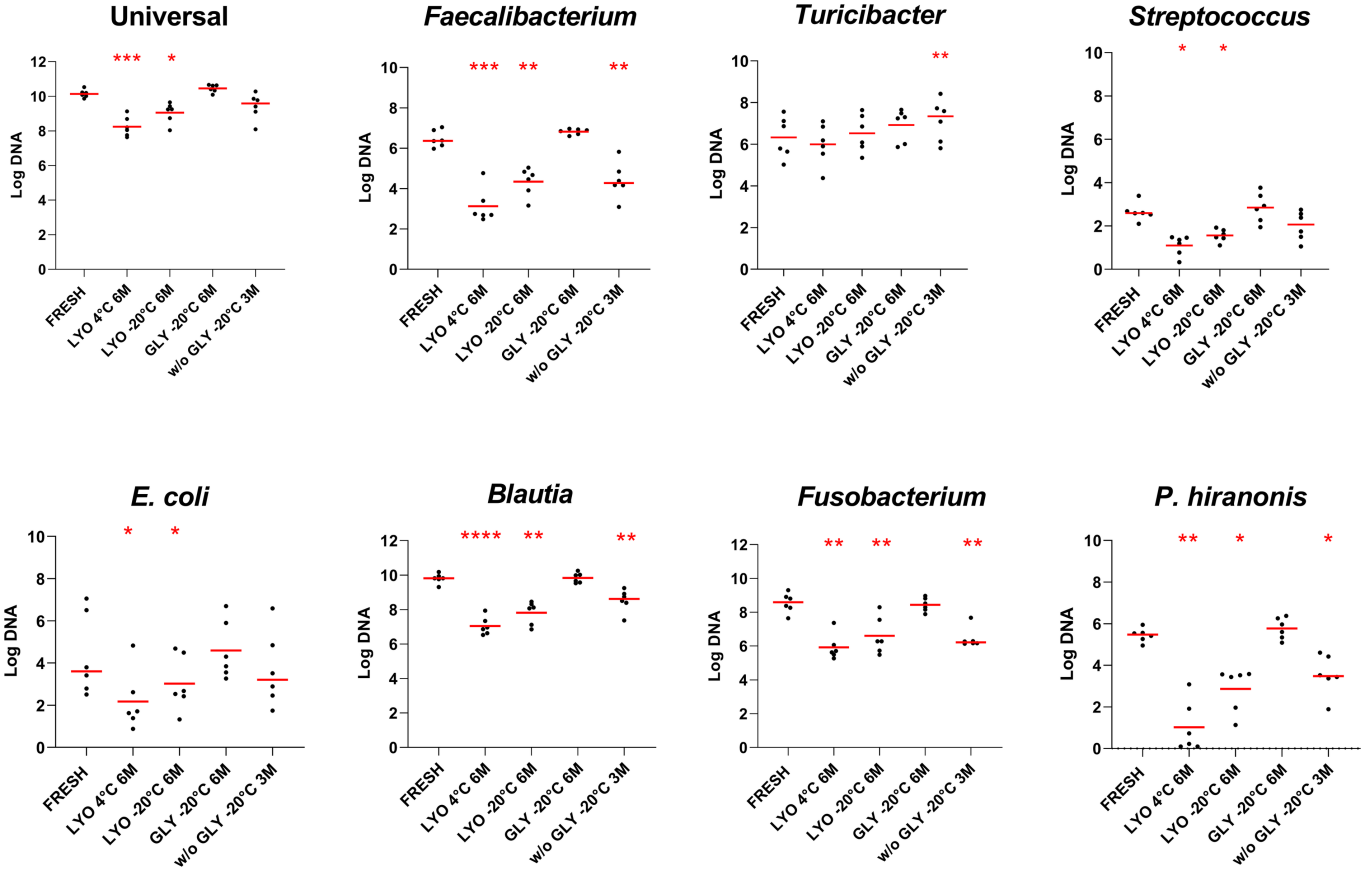
Bacterial abundances assessed in fecal samples by PMA-qPCR at the last time point for each conservation method: lyophilization stored at 4°C (LYO 4°C 6 M) and −20°C (LYO –20°C 6 M), and freezing with glycerol (GLY –20°C 6 M) and without cryoprotectant stored at −20°C (w/o GLY –20°C 3 M). The red line indicates the median value for each evaluated condition. The *p*-values are denoted as follows: * for *p* ≤ 0.05, ** for *p* ≤ 0.01, *** for *p* ≤ 0.001, and **** for *p* ≤ 0.0001.

Using PMA-qPCR to assess bacterial viability, abundances of *Faecalibacterium*, *E. coli*, *Streptococcus*, *Blautia*, *Fusobacterium,* and *P. hiranonis* reduced significantly in lyophilized samples stored at both 4°C and −20°C after 6 months (*p* < 0.05). In frozen feces without glycerol, only *Streptococcus* and *E. coli* were not significantly reduced after 3 months (*p* > 0.05). No statistically significant differences were observed in glycerol-preserved samples for all assessed bacteria taxa over 6 months of storage (*p* > 0.05) (Table 2 and Figure 1). Detailed results for all tested bacterial taxa in each one of the conditions over time can be found in Supplementary Figure S2.

**Table 2:** Bacterial abundances, expressed in log DNA, assessed in fecal samples by PMA-qPCR at the last time point for each conservation method: lyophilized fecal samples stored at 4°C and −20°C, and freezing with glycerol and without cryoprotectants stored at −20°C.

	Fresh fecal sample	LYO 4°C 6M	LYO –20°C 6M	GLY –20°C 6M	w/o GLY –20°C 3M
	Median (Range)	Median (Range) Adjusted *p*-value	Median (Range) Adjusted *p*-value	Median (Range) Adjusted *p*-value	Median (Range) Adjusted *p*-value
Universal	10.14 (9.87–10.53)	8.13 (7.64–9.12) 0.0010	9.25 (8.04–9.65) 0.0260	10.51 (10.09–10.66) 0.3285	9.58 (8.09–10.28) 0.1704
*Faecalibacterium*	6.37 (5.97–7.04)	2.72 (2.48–4.77) 0.0003	4.57 (3.16–5.04) 0.0037	6.87 (6.60–6.97) 0.1701	4.28 (3.09–5.83) 0.0021
*Turicibacter*	6.33 (5.02–7.56)	6.04 (4.37–7.10) 0.5354	6.47 (5.34–7.64) 0.8720	7.27 (5.87–7.65) 0.1884	7.34 (5.81–8.42) 0.0040
*Streptococcus*	2.59 (2.09–3.39)	1.27 (0.33–1.48) 0.0171	1.55 (1.10–1.93) 0.0135	2.85 (1.94–3.76) 0.7315	2.06 (1.05–2.75) 0.1051
*E. coli*	3.61 (2.51–7.06)	1.67 (0.88–4.83) 0.0185	2.60 (1.32–4.68) 0.0313	4.07 (3.26–6.70) 0.8319	3.20 (1.74–6.59) 0.2908
*Blautia*	9.82 (9.30–10.17)	6.91 (6.53–7.94) <0.0001	8.09 (6.85–8.45) 0.0054	9.82 (9.53–10.25) 0.9976	8.62 (7.37–9.24) 0.0017
*Fusobacterium*	8.59 (7.65–9.31)	5.67 (5.27–7.37) 0.0012	6.28 (5.48–8.30) 0.0083	8.41 (7.89–8.97) 0.8871	6.22 (6.13–7.67) 0.0028
*P. hiranonis*	5.47 (4.96–5.94)	0.47 (0.10–3.08) 0.0015	3.48 (1.13–3.58) 0.0121	5.78 (5.08–6.37) 0.1425	3.48 (1.89–4.61) 0.0140

The *p*-values were calculated by comparing the last time point for each conservation method with bacterial abundances assessed in fresh fecal samples (first column). Adjusted *p*-values from Dunnett’s multiple comparison tests were reported. LYO, lyophilized feces; 3M, 3 months; 6M, 6 months; GLY, feces preserved with glycerol; w/o GLY, feces conserved without cryoprotectants.

### 3.2 Quantification of *Peptacetobacter hiranonis* by bacterial culture

**Figure 2:**
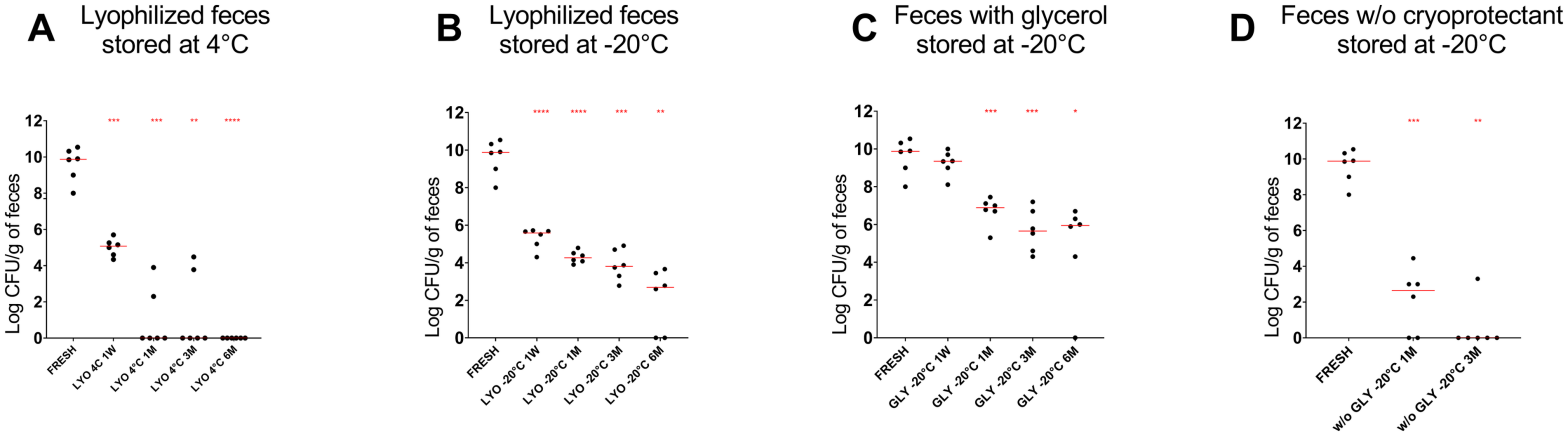
The abundance of *P. hiranonis* in feces assessed by culture at different time points: fresh, after 1 week of storage, and after 1, 3, and 6 months of storage. The abundance of *P. hiranonis* is expressed in log CFU/g of feces. The evaluated conservation methods were lyophilization stored at 4°C **(A)** and −20°C **(B)**, and freezing with glycerol **(C)** and without cryoprotectant **(D)** stored at −20°C. LYO, lyophilized feces; 1W, 1 week; 1M, 1 month; 3M, 3 months; 6M, 6 months; GLY: feces conserved with glycerol; w/o GLY: feces conserved without cryoprotectant. The red lines indicate the median value for each evaluated condition. The *p*-values are denoted as follows: * for *p* ≤ 0.05, ** for *p* ≤ 0.01, *** for *p* ≤ 0.001, and **** for *p* ≤ 0.0001.

A decrease in the abundance of *P. hiranonis* was observed by culture across all tested conditions (*p* < 0.0001). After 6 months of storage, the abundance of *P. hiranonis* was lower in lyophilized samples stored at both 4°C [median (range): 0 (0–0) log colony-forming unit (CFU)/g] compared to samples stored at −20°C [2.69 (0–3.66)]. Additionally, *P. hiranonis* abundance was higher in glycerol-preserved samples [5.94 (0–6.70)] after 6 months, as compared to frozen samples without cryoprotectants [0 (0–3.30)] after 3 months (Figure 2).

### 3.3 Comparison between *Peptacetobacter hiranonis* quantification by PMA-qPCR and bacterial culture

**Figure 3:**
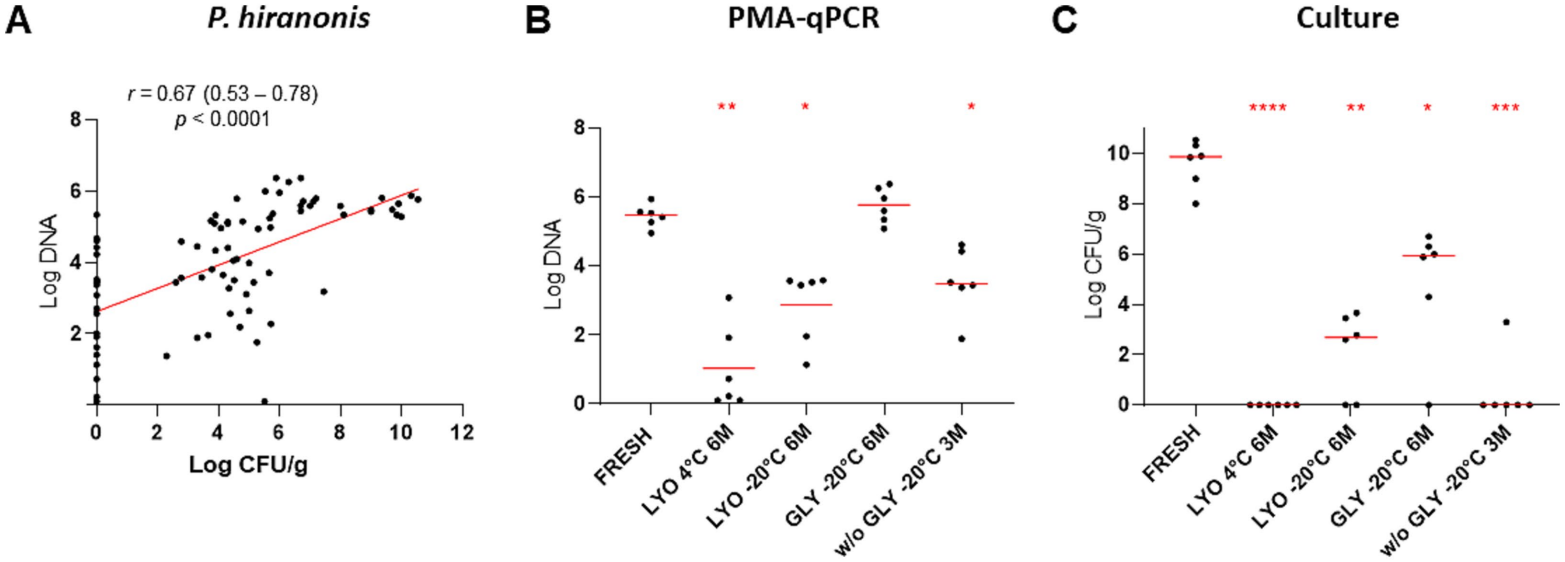
Comparison between the quantification of *P. hiranonis* by PMA-qPCR, expressed in log DNA, and bacterial culture, expressed in log CFU/g of feces. **(A)** Spearman’s correlation between the abundance of *P. hiranonis* quantified by PMA-qPCR and culture. Spearman’s correlation coefficient (*r*), 95% confidence interval, and *p*-value were provided. **(B)** Abundance of *P. hiranonis* in lyophilized fecal samples stored at 4°C and −20°C, glycerol-preserved fecal samples, and fecal samples preserved without cryoprotectant stored at −20°C assessed by PMA-qPCR at the last time point for each conservation method. **(C)** Abundance of *P. hiranonis* in lyophilized samples stored at 4°C and −20°C, glycerol-preserved fecal samples, and fecal samples preserved without cryoprotectant stored at −20°C assessed by culture at the last time point for each conservation method. LYO, lyophilized feces; GLY, feces conserved with glycerol; w/o GLY, feces conserved without cryoprotectant; 3M, 3 months; 6M, 6 months. The *p*-values are denoted as follows: * for *p* ≤ 0.05, ** for *p* ≤ 0.01, *** for *p* ≤ 0.001, and **** for *p* ≤ 0.0001.

Using PMA-qPCR to assess bacterial viability, abundance of *P. hiranonis* was reduced in lyophilized samples kept at 4°C and −20°C after 6 months and in frozen samples without glycerol after 3 months (*p* < 0.05). Moreover, decreased *P. hiranonis* abundance was observed for all tested conditions by culture (*p* < 0.05) (Figure 3 and Table 3). Correlation between PMA-qPCR and *P. hiranonis* viability assessed by cultured demonstrate a positive correlation Spearman *r* = 0.6732 (*p* < 0.0001), being affected by several samples (17/84, 20%) which *P. hiranonis* was not recovered by culture but detected by PMA-qPCR.

**Table 3:** The abundance of *P. hiranonis*, expressed in log CFU/g of feces, assessed in fecal samples by culture at the last time point for each conservation method: lyophilized feces stored at 4°C and −20°C, and freezing with glycerol and without cryoprotectants stored at −20°C.

Conservation methods	*P. hiranonis* (log CFU/g of feces)	
	Median (range)	Adjusted *p*-value
Fresh fecal sample	9.87 (8–10.54)	-
Lyophilized fecal samples kept at 4°C 6M	0 (0–0)	<0.0001
Lyophilized fecal samples kept at −20°C 6M	2.69 (0–3.66)	0.0016
Glycerol-preserved fecal samples kept at −20°C 6M	5.94 (0–6.70)	0.0192
Fecal samples kept at −20°C 3M	0 (0–3.30)	0.0002

The *p*-values were calculated by comparing the last time point for each conservation method to bacterial abundances assessed in fresh fecal samples (first row). Adjusted p-values from Dunnett’s multiple comparison tests were reported. 3M, 3 months; 6M, 6 months.

## 4 Discussion

In this study, we assessed the overall reduction in viability of several bacteria in healthy canine feces under different conservation methods. The bacterial viability was evaluated in feces stored at −20°C with and without the addition of glycerol and in lyophilized feces stored at 4°C and −20°C for up to 6 months. Storage at −20°C seems more adequate for preservation of bacterial viability compared to storage at 4°C. The bacteria quantified included *E. coli, Faecalibacterium, Blautia, P. hiranonis, Fusobacterium*, *Turicibacter,* and *Streptococcus,* which were evaluated through PMA-qPCR. In addition to viability assessment by PMA-qPCR, *P. hiranonis* viability was also quantified through bacterial culture, with both methods indicating a reduction in the viability of *P. hiranonis* across time points.

In veterinary medicine, there is a lack of information regarding bacterial viability in fecal samples intended for FMT use (Chaitman and Gaschen, 2021; Collier et al., 2022; Papanicolas et al., 2019). Guidelines for FMT in veterinary medicine have been recently published, however, the screening criteria and analysis of the donor microbiota are not consistent across veterinary clinics (Chaitman and Gaschen, 2021; Winston et al., 2024). Clinical response is the ultimate outcome to evaluate the efficacy of FMT preparations, however, testing each small protocol variation in clinical trials is not feasible. While it is currently unknown whether live bacteria are required for FMT to result in clinical response and to restore gut microbiota functionality, which confer gut health benefits (Khoruts and Sadowsky, 2016), it is reasonable to ensure that specific keystone bacteria remain viable for their beneficial effects. In particular, re-colonization with *P. hiranonis*, which plays an essential role in bile acid metabolism (Ridlon et al., 2020; Kitahara et al., 2001) and is commonly missing in severe dysbiosis, is a desired outcome of FMT (Chaitman et al., 2020; Toresson et al., 2023). Additionally, the bacterial taxa *Faecalibacterium, Fusobacterium, Turicibacter,* and *Blautia* display a key role in the production of SCFAs and have been correlated to the maintenance of a healthy intestinal microbiome (Vital et al., 2014; Liu et al., 2021; Lynch et al., 2023; Miquel et al., 2013).

Metabolites, such as bile acids, amino acids, short and long-chain fatty acids, vitamins, and polysaccharides, are transplanted in association with bacteria in FMT, however different methods of FMT preparations (e.g., freezing, freeze-drying, and not frequently used, dehydration) might affect metabolite concentration in the fecal content (Reygner et al., 2020; Martinez-Gili et al., 2020). In this study, we have not evaluated the effect of FMT preparation methods on the fecal metabolites, although, previous reports have shown limited or no effects on SCFA (Reygner et al., 2020) or bile acid concentration (unpublished data). Fecal filtrated transplant (FFT), lacking viable bacteria but containing bacterial debris and metabolites, has been described as an alternative to FMT, since FMT may represent a risk for immunocompromised patients when screening methods for FMT donors fail to detect potential risks, i.e., pathogens. In addition, the presence of bacteriophages has been linked with beneficial effects observed after FFT in humans and animal models (Ott et al., 2017; Brunse et al., 2021). In summary, the FMT mechanisms are complex, and clinical effectiveness may be associated with different factors from live bacteria to metabolites.

While few studies describe bacterial viability (Papanicolas et al., 2019; Barko et al., 2024), most relied on PMA-qPCR for assessment rather than traditional bacterial cultivation methods. Although PMA-qPCR is a well-established method to assess bacterial viability, it is important to consider limitations related to false-positive results, since PMA will allow the detection of viable-non-culturable bacteria by qPCR (van Frankenhuyzen et al., 2011; Wolffs et al., 2005). In our study, we evaluated the viability of *P. hiranonis* through bacterial culture, and in a substantial fraction of the samples (17/84, 20%), *P. hiranonis* was detected by PMA-qPCR, but not by culture. Parallel to limitations associated with PMA, *P. hiranonis* is considered a fastidious bacterium and false-negative results may be observed. However, it is important to note that live *P. hiranonis* is needed for colonization and further conversion of bile acids. We focused solely on *P. hiranonis,* a strictly anaerobic bacterium highly sensitive to oxygen exposure (Chen et al., 2020; Kitahara et al., 2001). However, assessing *P. hiranonis* viability through culture may serve as a proxy for other anaerobic bacteria, considering their shared characteristic of sensitivity to oxygen exposure (see Supplementary Figure S3).

Sequencing methods are commonly applied to evaluate microbial composition in fecal samples. The quantification of bacteria in different taxonomic levels is expressed in relative abundance, and the generated data are treated as compositional (Gloor et al., 2017). In our study, a target qPCR method was used for absolute quantification of core bacterial genera and species present in the intestinal microbiome of dogs. In the article published by Barko et al. (2024), long-read 16S rRNA amplicon sequencing was used, and relative abundances of *Turicibacter sanguinis*, *Streptococcus pasteuri,* and *Blautia glucerasea* were found to increase over time in samples cryopreserved in glycerol and stored at −20°C. In contrast, our results indicated no significant difference in the absolute abundance of *Turicibacter* spp., *Streptococcus* spp., or *Blautia* spp. in glycerol-preserved samples stored at −20°C.

Targeted methods, such as qPCR, are more reliable for the quantification of bacterial absolute abundance compared to sequencing methods (Wang et al., 2021), which are known for presenting variable results affected by sample collection method, storage, DNA extraction, and sequencing method, and further by the bioinformatics tools applied (Tourlousse et al., 2021). In addition, considering the limitations of the PMA-qPCR methodology previously mentioned, more studies are needed to define whether the observed increase or decrease in the abundances of *Turicibacter* and *Blautia* reflects the abundance of live bacteria in FMT preparations over time and across different storage temperatures. *Turicibacter* and *Blautia* are Gram-positive strictly anaerobic bacteria capable of growth between 30 and 45°C. An observed increase in abundance over time may indicate PMA saturation, a scenario where the amount of PMA used is insufficient to bind all DNA from non-viable or membrane-damaged cells (Liu et al., 2021; Maki and Looft, 2022).

A decreased relative abundance of *Fusobacterium* spp. over time was reported in samples kept at −20°C with glycerol using long-read 16S rRNA amplicon sequencing (Barko et al., 2024). However, our target qPCR did not detect a reduction in *Fusobacterium* preserved with glycerol at −20°C, only in lyophilized feces stored at 4°C and −20°C and feces preserved without cryoprotectant stored at −20°C. Finally, while Barko et al. (2024) reported an increased relative abundance of *P. hiranonis* over time in glycerol-preserved samples, our study found the absolute abundance of *P. hiranonis* unchanged by PMA-qPCR or decreased by culture in fecal samples after 6 months of storage (see Figures 1, 2). Differences between data reported by Barko and this current publication may be explained by the different methodologies applied: qPCR and sequencing. It is important to mention that sequencing methods have inherent limitations in terms of quantification, repeatability, and reproducibility; therefore, results need to be evaluated carefully and, when possible, confirmed by targeted assays.

The processing methods employed in this study require a homogenization step, exposing the fecal sample to ambient air and consequently to high levels of oxygen. Homogenization and filtration are required steps in several consensus guidelines for FMT preparations in humans (Cammarota et al., 2017; Mullish et al., 2018) and in veterinary medicine (Chaitman and Gaschen, 2021). While the deleterious effects of oxygen exposure can be mitigated when processing the samples in an anaerobic environment (i.e., anaerobic chamber), it is important to note that ensuring an oxygen-free environment requires specialized and trained personnel and specific equipment, potentially increasing operational costs for the FMT preparation and rendering it inaccessible to clinicians outside of referral centers. Though more studies are needed to clarify if the exposure to oxygen during preparation could be detrimental to FMT effectivity, our goal was to analyze viability in preparation methods that were commonly used and easily applicable in clinical practice without the need for specialized equipment, i.e., preparation in the presence of oxygen, use of glycerol as a cryopreservative, and storage at −20°C.

Besides the effect of oxygen exposure, different storage temperatures may affect bacterial viability in long-term storage. The temperatures used in this study reflect the temperature available in most veterinary practices (refrigeration at 4°C and freezing with household-grade freezers at –20°C), because −80°C freezers are usually not available outside of laboratory facilities. Costello et al. reported that long-term storage of FMT preparation, more than two months, preserved with glycerol at –80°C presented satisfactory results when used to prevent *C. difficile* recurrence (Costello et al., 2015). In another study published by Lee et al., fresh and frozen stored at –20°C feces were compared leading to similar outcomes – recovery from *C. difficile* infection (Lee et al., 2016). Although there is a lack of similar studies in the veterinary field, research in humans points out that similar outcomes are accomplished when using fresh and frozen feces for FMT. Therefore, in this study we chose to test storage at -20°C to reflect the current use of FMT preparation methodologies in veterinary clinics, making our results translatable to clinical practice.

*Faecalibacterium, Blautia, Turicibacter,* and *Fusobacterium* are involved in SCFA production, a metabolite that is crucial for nourishing colonocytes (Vital et al., 2014; Liu et al., 2021; Lynch et al., 2023; Miquel et al., 2013; Hosomi et al., 2022). These bacterial genera are strict anaerobes and are among the most abundant bacteria taxa in the gut microbiota of dogs and cats (AlShawaqfeh et al., 2017; Sung et al., 2023; Sung et al., 2022). Dysbiosis often leads to a reduction in the abundance of strictly anaerobic bacteria, accompanied by an increase in facultative anaerobes such as *E. coli* and *Streptococcus* (AlShawaqfeh et al., 2017; Pilla et al., 2020; Guard et al., 2019; Sung et al., 2022; Rivera-Chavez et al., 2017). Although the precise mechanism by which the resident microbiota limits pathogen expansion remains unclear (Deleu et al., 2021), SCFA appears to play an important role in maintaining gut homeostasis, intestinal barrier integrity, as well as overall metabolism and energy balance (Portincasa et al., 2022; Ma et al., 2022). Therefore, the survival of these SCFA-producing taxa in FMT preparations may directly impact FMT outcomes.

While frozen feces preserved with glycerol presented better results in conserving *P. hiranonis* viability, lyophilization is an alternative methodology for FMT pill manufacture, given that most capsules dissolve upon contact with water or humidity. The absence of water in lyophilized feces during the manufacture of FMT capsules ensures the preservation of the capsules’ structure. Lyophilization can be performed with or without cryoprotectants (Zain et al., 2022). In our study, we have chosen to perform the lyophilization without any cryoprotectants, as this aligns with a more easily replicable method in veterinary practice facilities. However, further research is necessary to elucidate the effect of cryoprotectants on bacterial viability in lyophilized feces.

Similar to a previous study, while there was a reduction of potentially beneficial bacteria, facultative anaerobic bacteria, i.e., *E. coli* and *Streptococcus*, assessed in this study did not increase over time (Papanicolas et al., 2019). Increased *Enterobacteriaceae* abundance in the intestine is associated with an inflammatory response and it has been linked to inflammatory bowel disease, type 2 diabetes, and colorectal cancer in humans (Baldelli et al., 2021), as well as chronic enteropathy and antibiotic-induced dysbiosis in dogs and cats (AlShawaqfeh et al., 2017; Giaretta et al., 2018; Blake et al., 2019; Guard et al., 2019; Sung et al., 2022). In addition, elevated *Streptococcus* abundance has been correlated to antibiotic-induced dysbiosis and chronic enteropathy in dogs and cats (Chaitman et al., 2020; Pilla et al., 2020; Sung et al., 2022), highlighting the role of increased oxygen levels in the intestinal lumen as a potent cause and trigger for inflammation and severe dysbiosis (Rivera-Chavez et al., 2017). Moreover, gastrointestinal dysmotility and malabsorption have been associated with the overgrowth of facultative anaerobic bacterial species, including increased *E. coli* and *Streptococcus* (Bala et al., 2006), where impaired fat absorption and motility might contribute to intestinal inflammation (Singh et al., 2021).

Among the limitations of our study, we were only able to analyze frozen feces without cryoprotectants for a period of up to 3 months. Unfortunately, there were not enough sample aliquots available for further evaluation. Additionally, other cryoprotectants described in the literature were not assessed in this study (Hubalek, 2003), as well as additional storage temperatures, such as −80°C or controlled room temperature (20–25°C). However, lyophilized feces and frozen feces with and without glycerol for preservation are the most common methods used for FMT preparation in veterinary medicine and are considered more accessible for veterinarians (Toresson et al., 2023). Finally, another important limitation is associated with the use of the PMA-qPCR methodology, which may yield false-positive results (Miquel et al., 2013; van Frankenhuyzen et al., 2011). False positive results using PMA-qPCR are linked to incomplete inhibition of amplification of free-floating DNA due to insufficient PMA in a sample rich in free-floating DNA. In addition, dead bacterial cells with intact cell membranes cannot be differentiated from viable cells by PMA-qPCR. Finally, the small sample size evaluated in this study reduces the statistical power, and further studies containing larger sample sizes are needed.

In summary, a reduction of *Faecalibacterium*, *E. coli*, *Streptococcus*, *Blautia*, *Fusobacterium,* and *P. hiranonis* abundances was observed by culture and or PMA-qPCR across the time points evaluated. Better conservation of bacterial viability can be achieved by freezing fecal samples with glycerol. Despite decreases in viability, lyophilized feces stored for up to 6 months at −20°C may represent a valid alternative for manufacturing FMT capsules. Finally, clinical studies are needed to elucidate how these differences in bacterial viability affect FMT outcomes.

## Data Availability

The raw data supporting the conclusions of this article will be made available by the authors, without undue reservation.
