# Supplementary material for: Prolonged storage reduces viability of Peptacetobacter (Clostridium) hiranonis and core intestinal bacteria in fecal microbiota transplantation preparations for dogs
Source: Front Microbiol. 2025 Jan 7;15:1502452. doi: 10.3389/fmicb.2024.1502452 (PMC11747423; doi:10.3389/fmicb.2024.1502452)
Supplement: Supplementary file 1 [file Data_Sheet_1.docx]

Prolonged Storage Reduces Viability of *Peptacetobacter (Clostridium) hiranonis* and Core Intestinal Bacteria in Fecal Microbiota Transplantation Preparations for Dogs

Bruna Correa Lopes^1*^, Jonathan Turck^1^, M Katherine Tolbert^1^, Paula R Giaretta^1^, Jan S Suchodolski^1^, Rachel Pilla^1,2^

^1^Gastrointestinal Laboratory, Department of Small Animal Clinical Sciences, Texas A&M University, College Station, TX, USA

^2^Department of Veterinary Pathology, Hygiene and Public Health, University of Milan, Italy

*** Correspondence:**Bruna Correa Lopes
brunalopes@tamu.edu

Keywords: *Clostridium hiranonis*, dysbiosis, dog, cryoprotectant, PMA, bacterial culture, lyophilization, bile acid metabolism.

**Supplementary Table 1.** Descriptive information on clinically healthy dogs undergoing screening as potential donors of feces for fecal microbiota transplantation.

|  | **Breed** | **Sex** | **Age** | **Weight (kg)** |
| --- | --- | --- | --- | --- |
| **1** | Golden Retriever | Spayed Female | 2 | 29.6 |
| **2** | Golden Retriever | Castrated Male | 7 | 39.2 |
| **3** | Rhodesian Ridgeback | Male | 4 | 41.0 |
| **4** | Rhodesian Ridgeback | Female | 2 | 36.4 |
| **5** | Rhodesian Ridgeback | Spayed Female | 6 | 30.0 |
| **6** | Australian Shepherd | Castrated Male | 6 | 24.8 |

**Supplementary Table 2.** Forward (F) and reverse (R) primers used in this study as described by AlShawaqfeh *et al*. (2017) and Sung *et al*. (2022)

|  | **Sequence (5΄- 3΄)** | **Target** |
| --- | --- | --- |
| F | GAAGGCGGCCTACTGGGCAC | *Faecalibacterium* |
| R | GTGCAGGCGAGTTGCAGCCT |  |
| F | KGGGCTCAACMCMGTATTGCGT | *Fusobacterium* |
| R | TCGCGTTAGCTTGGGCGCTG |  |
| F | TCTGATGTGAAAGGCTGGGGCTTA | *Blautia* |
| R | GGCTTAGCCACCCGACACCTA |  |
| F | CCTACGGGAGGCAGCAGT | Universal |
| R | ATTACCGCGGCTGCTGG |  |
| F | CAGACGGGGACAACGATTGGA | *Turicibacter* |
| R | TACGCATCGTCGCCTTGGTA |  |
| F | GTTAATACCTTTGCTCATTGA | *Escherichia coli* |
| R | ACCAGGGTATCTAATCCTGTT |  |
| F | AGTAAGCTCCTGATACTGTCT | *Peptacetobacter (Clostridium) hiranonis* |
| R | AGGGAAAGAGGAGATTAGTCC |  |
| F | TTATTTGAAAGGGGCAATTGCT | *Streptococcus* |
| R | GTGAACTTTCCACTCTCACAC |  |

**Supplementary Figure 1.** *Peptacetobacter (Clostridium) hiranonis* colony morphology. A: Mixed bacterial growth from a fecal sample inoculated into a Brucella blood agar plate incubated at 37°C in anaerobiosis for 48 hours. Colonies of *P. hiranonis* confirmed by qPCR are indicated by a yellow circle. B. Pure growth of *P. hiranonis* inoculated into a Brucella blood agar plate incubated at 37°C in anaerobiosis for 48 hours.


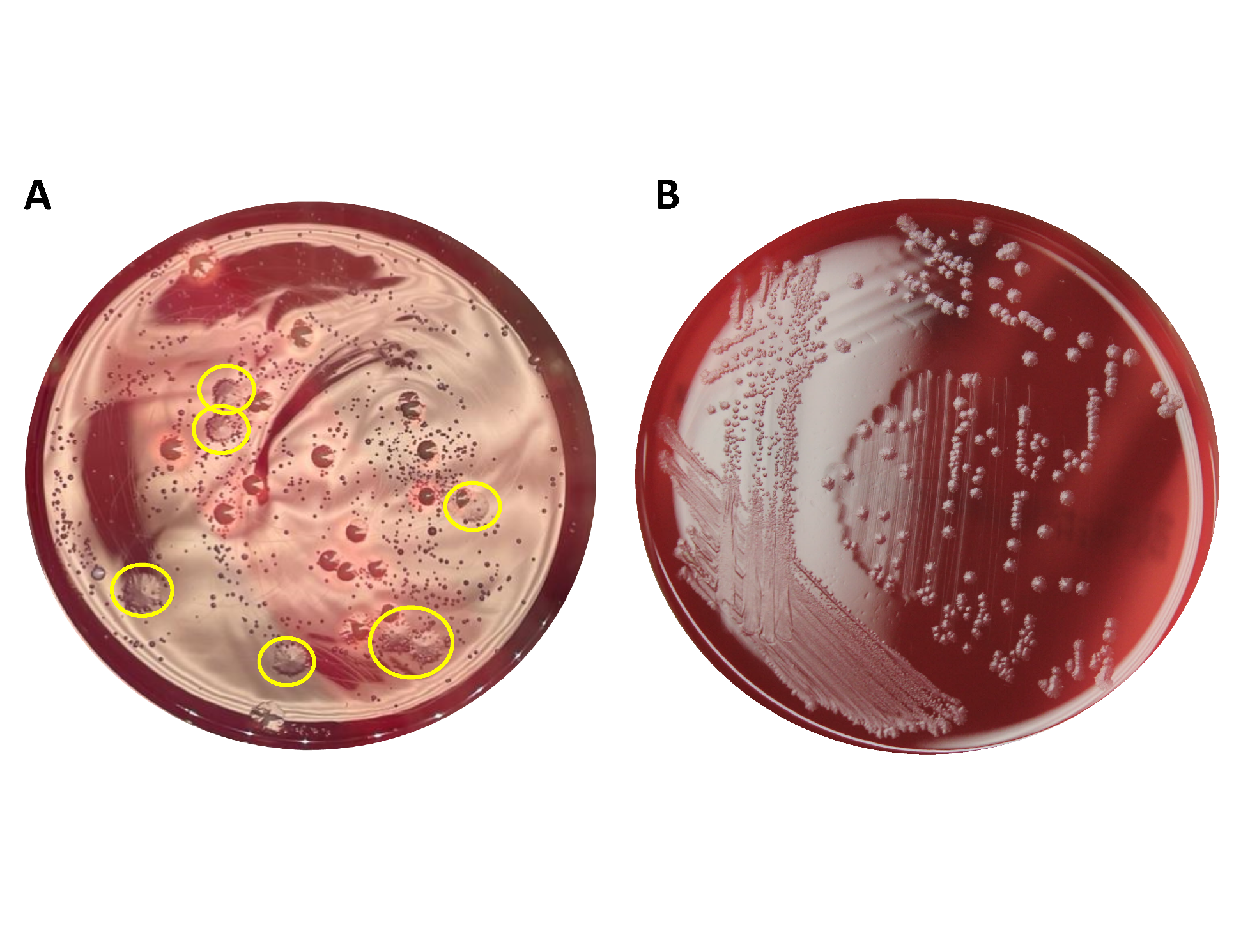


**Supplementary Figure 2.** Bacterial abundance assessed in fecal samples by PMA-qPCR at different time points for each conservation method: lyophilized fecal samples stored at 4°C (A) and -20°C (B), and freezing with glycerol (C) and without cryoprotectant (D) stored at -20°C.

**A**

**
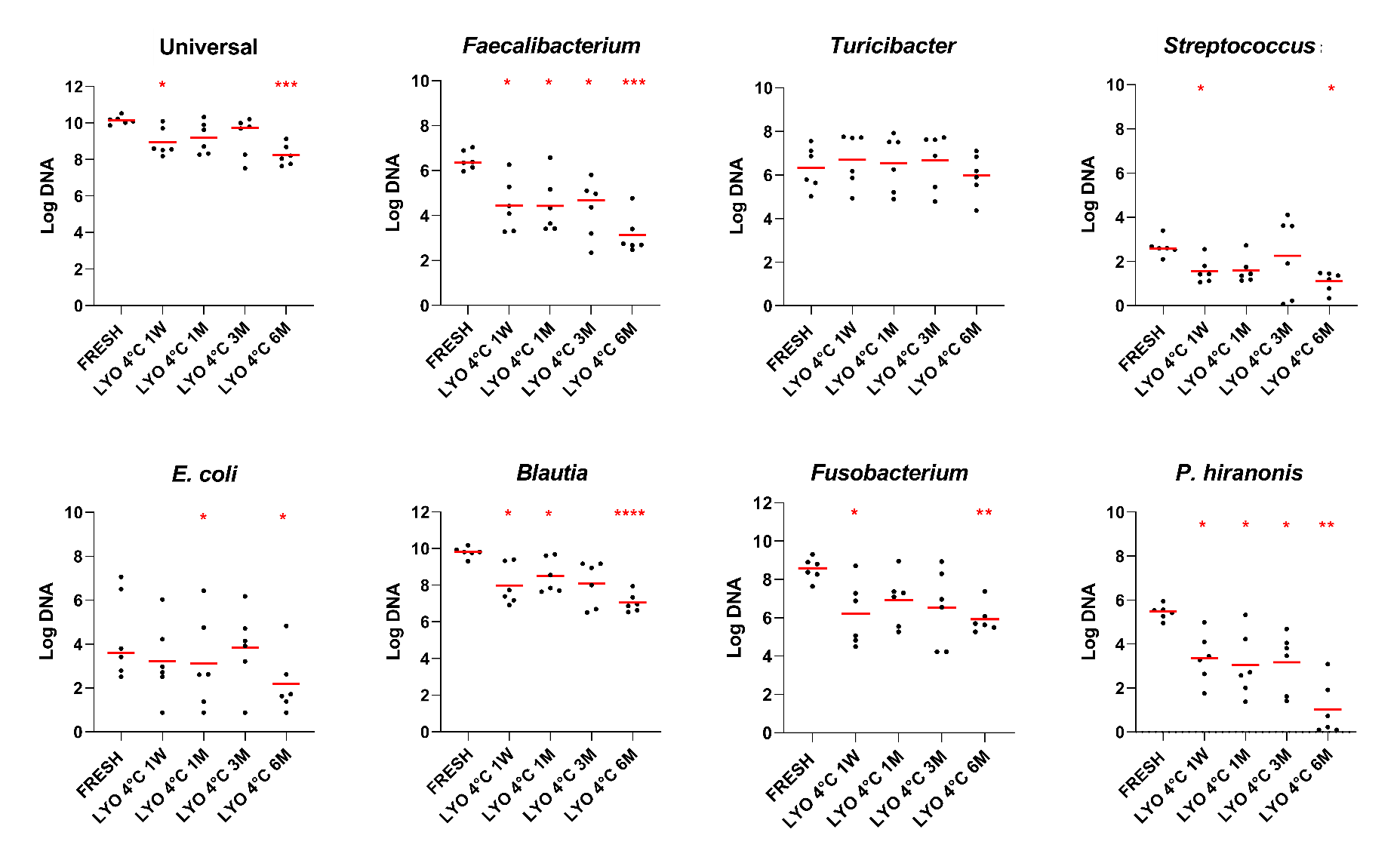
**

**B**

**
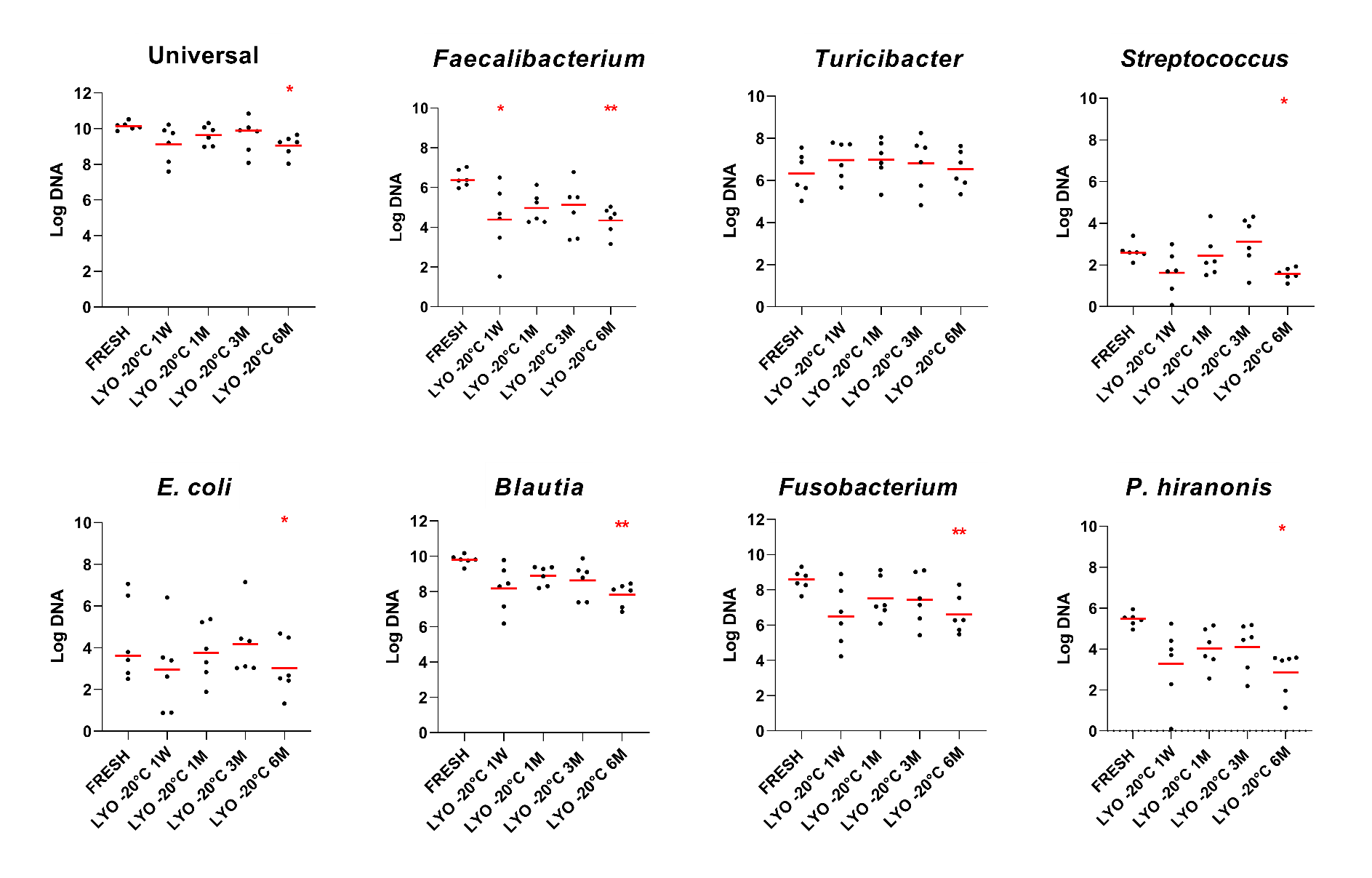
**

**C**

**
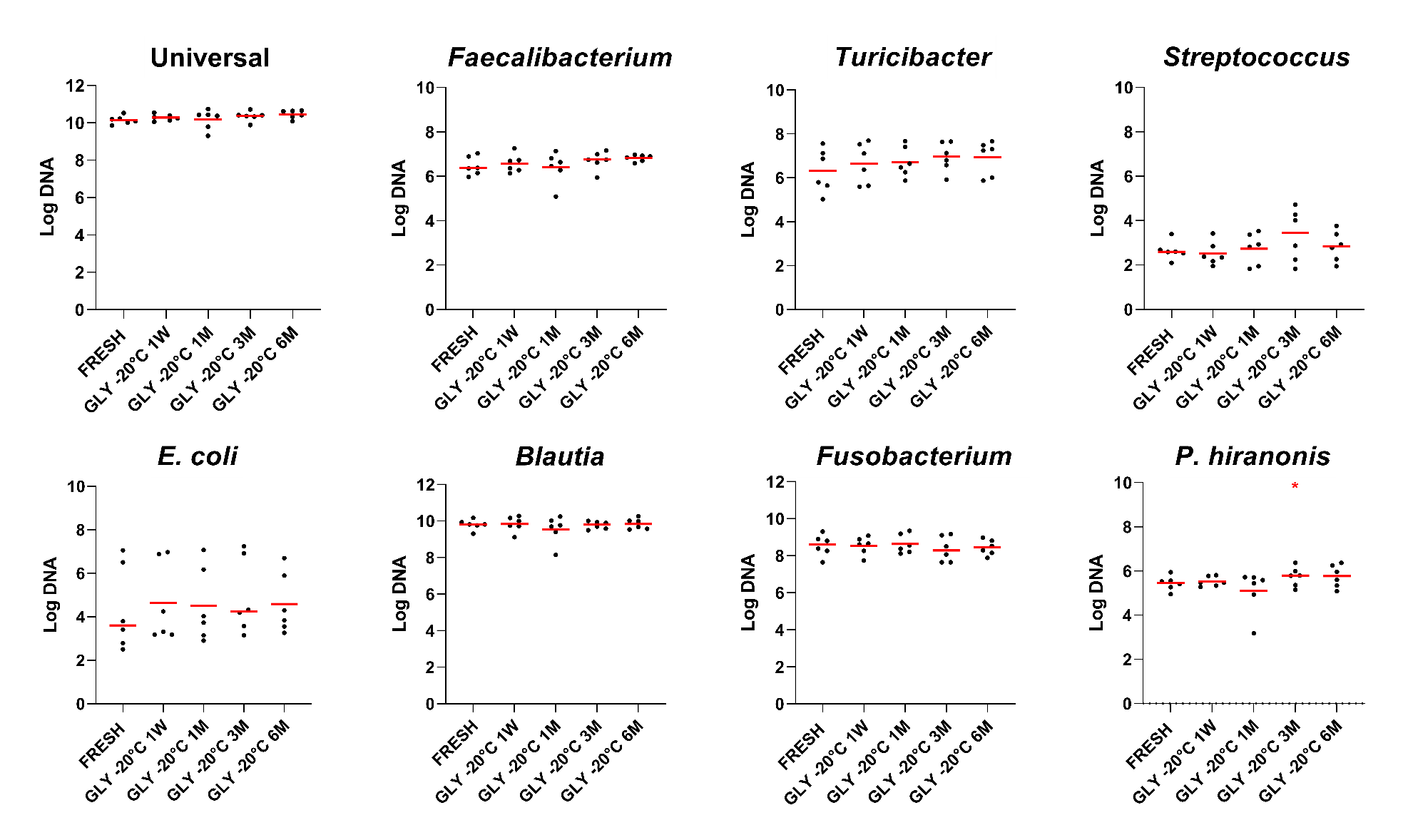
**

**D**

**
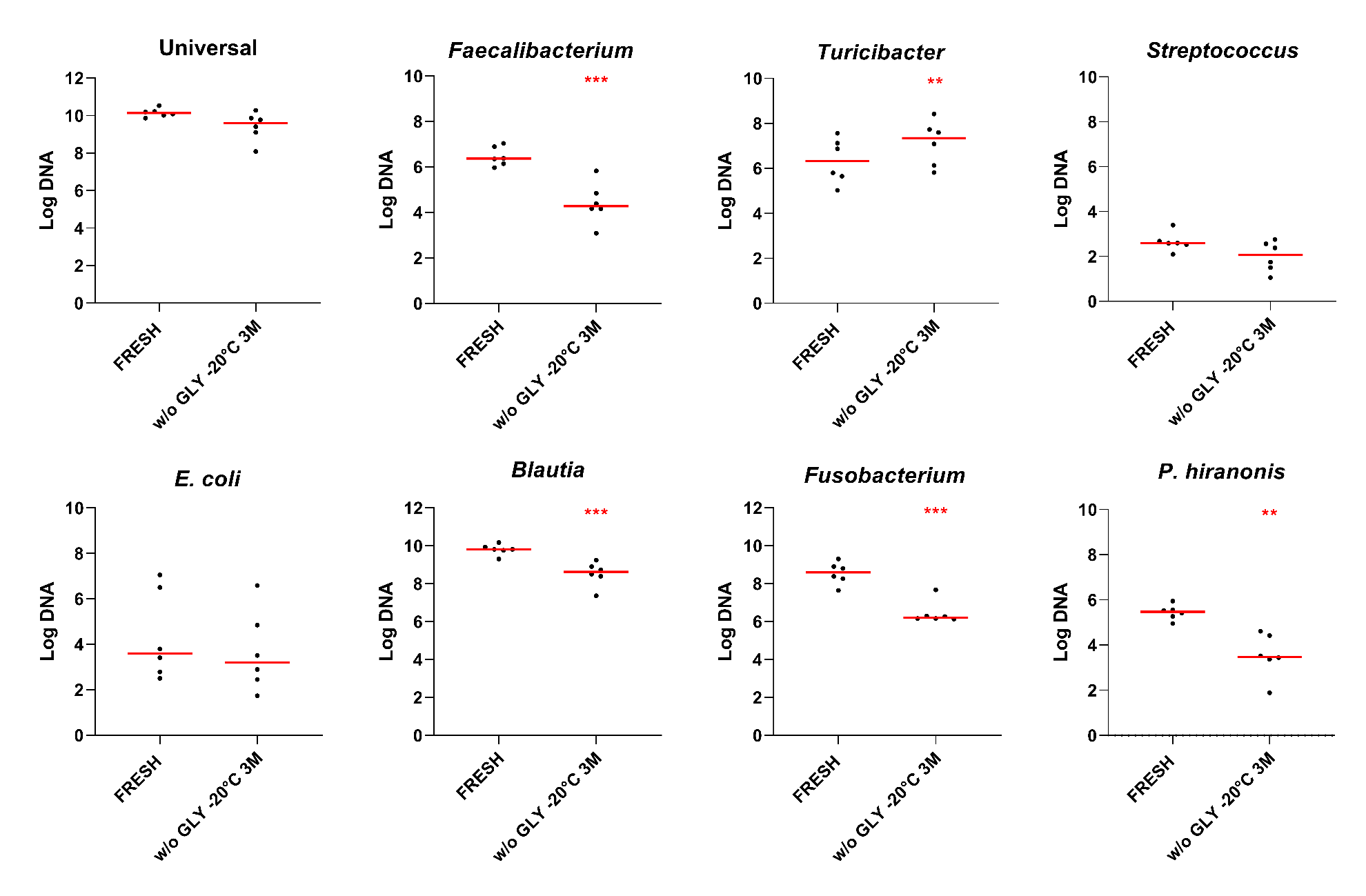
**

Note: LYO: lyophilized feces; 1W: one week; 1M: one month; 3M: three months; 6M: six months; GLY: feces conserved with glycerol; w/o GLY: feces conserved without cryoprotectant. The red lines indicate the median value for each evaluated condition. The *p*-values are denoted as follows: * for P ≤ 0.05, ** for P ≤ 0.01, *** for P ≤ 0.001, and **** for P ≤ 0.0001.

**Supplementary Figure 3.** Spearman’s correlation between the quantification of *P. hiranonis* by culture (n=84), expressed in log CFU/g of feces, and the quantification of *P. hiranonis*, *Blautia*, *Fusobacterium*, *Faecalibacterium*, and *Turicibacter* by PMA-qPCR, expressed by log DNA. Spearman’s correlation coefficient (*r*), 95% confidence interval, and P-values were provided in the graphs for all comparisons.

**
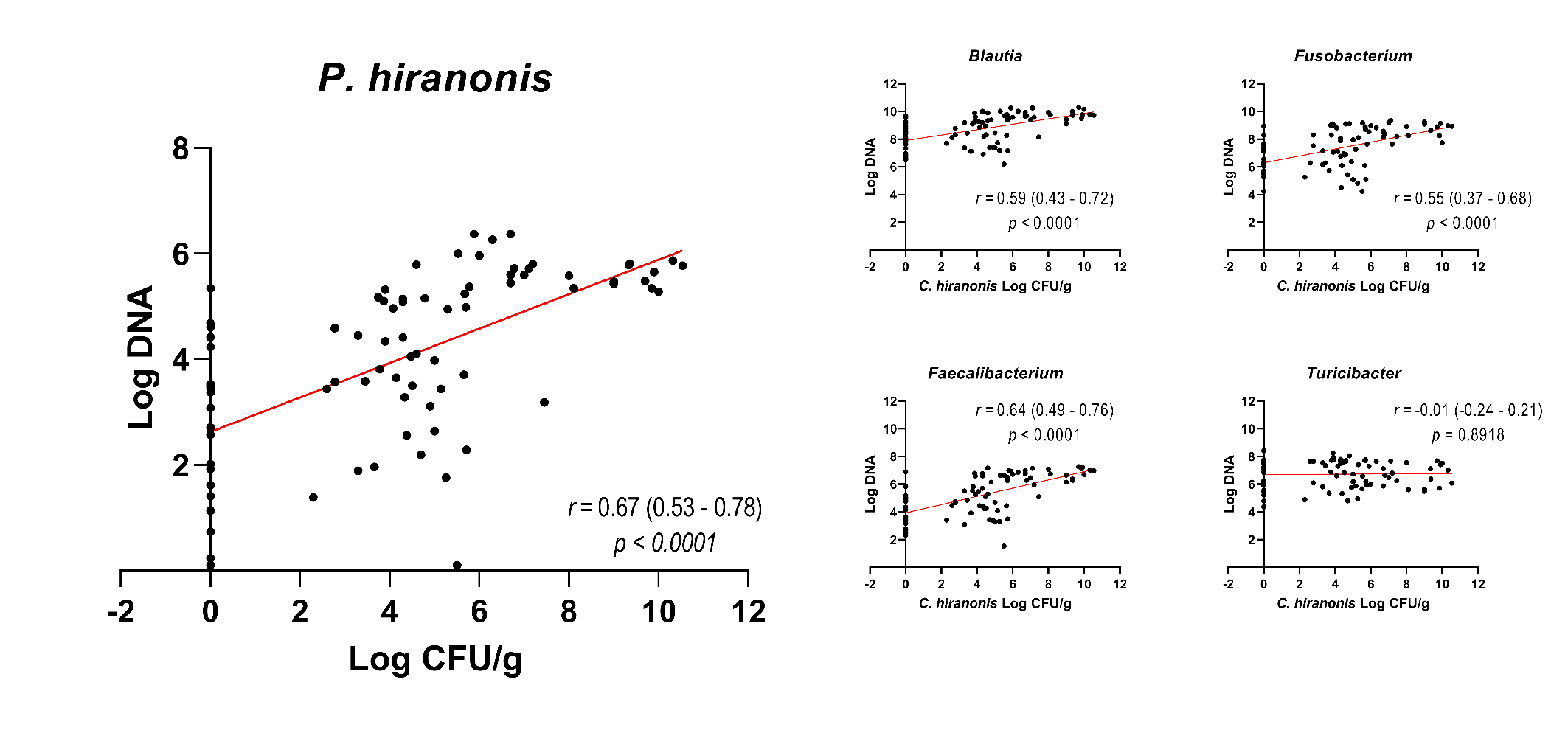
**
